# Colorectal cancer screening in newly insured Medicaid members: a review of concurrent federal and state policies

**DOI:** 10.1186/s12913-019-4113-2

**Published:** 2019-05-09

**Authors:** Meghan C. O’Leary, Kristen Hassmiller Lich, Yifan Gu, Stephanie B. Wheeler, Gloria D. Coronado, Sarah E. Bartelmann, Bonnie K. Lind, Maria E. Mayorga, Melinda M. Davis

**Affiliations:** 1 Department of Health Policy & Management, Gillings School of Global Public Health, University of North Carolina at Chapel Hill, 1105E McGavran-Greenberg Hall, Chapel Hill, NC 27599 USA; 2 Center for Health Systems Effectiveness, Oregon Health & Science University, Portland, OR USA; 3 Lineberger Comprehensive Cancer Center, University of North Carolina at Chapel Hill, Chapel Hill, NC USA; 4 Center for Health Promotion & Disease Prevention, University of North Carolina at Chapel Hill, Chapel Hill, NC USA; 5 Kaiser Permanente Center for Health Research, Portland, OR USA; 6 Center for Outcomes Research and Education, Portland, OR USA; 7 OHSU-PSU School of Public Health, Oregon Health & Science University, Portland, OR USA; 8 Edward P. Fitts Department of Industrial and Systems Engineering, North Carolina State University, Raleigh, NC USA; 9 Department of Family Medicine, Oregon Health & Science University, Portland, OR USA; 10 Oregon Rural Practice-based Research Network, Oregon Health & Science University, Portland, OR USA

**Keywords:** Colorectal Cancer, Health Policy, Health Promotion, Medicaid, Screening

## Abstract

**Background:**

Colorectal cancer (CRC) screening is underutilized by Medicaid enrollees and the uninsured. Multiple national and state policies were enacted from 2010 to 2014 to increase access to Medicaid and to promote CRC screening among Medicaid enrollees. We aimed to determine the impact of these policies on screening initiation among newly enrolled Oregon Medicaid beneficiaries age-eligible for CRC screening.

**Methods:**

We identified national and state policies affecting Medicaid coverage and preventive services in Oregon during 2010–2014. We used Oregon Medicaid claims data from 2010 to 2015 to conduct a cohort analysis of enrollees who turned 50 and became age-eligible for CRC screening (a prevention milestone, and an age at which guideline-concordant screening can be assessed within a single year) during each year from 2010 to 2014. We calculated risk ratios to assess whether first year of Medicaid enrollment and/or year turned 50 was associated with CRC screening initiation.

**Results:**

We identified 14,576 Oregon Medicaid enrollees who turned 50 during 2010–2014; 2429 (17%) completed CRC screening within 12 months after turning 50. Individuals newly enrolled in Medicaid in 2013 or 2014 were 1.58 and 1.31 times more likely, respectively, to initiate CRC screening than those enrolled by 2010. A primary care visit in the calendar year, having one or more chronic conditions, and being Hispanic was also associated with CRC screening initiation**.**

**Discussion:**

The increased uptake of CRC screening in 2013 and 2014 is associated with the timing of policies such as Medicaid expansion, enhanced federal matching for preventive services offered to Medicaid enrollees without cost sharing, and formation of Medicaid accountable care organizations, which included CRC screening as an incentivized quality metric.

**Electronic supplementary material:**

The online version of this article (10.1186/s12913-019-4113-2) contains supplementary material, which is available to authorized users.

## Background

Colorectal cancer (CRC) is the fourth most common type of cancer in the United States. Approximately 135,000 cases are newly diagnosed each year, representing 8% of all new cancer cases [1]. CRC is also the second leading cause of cancer-related deaths, accounting for an estimated 50,000 deaths annually [1, 2]. Over half of these CRC-specific deaths could be prevented by implementing interventions to improve timely screening, follow-up and treatment, and risk-factor modifications [3]. Multiple screening modalities, including colonoscopy and stool tests, are effective in reducing morbidity and mortality from CRC and are recommended for average-risk adults ages 50–75 by the U.S. Preventive Services Task Force [2]. Yet, screening rates remain relatively low, especially among adults newly age-eligible for CRC screening. In 2014, just 60.8% of all adults ages 50–64 were up-to-date with CRC screening nationally (median), compared to 76.1% of adults age 65 and older [4]. CRC screening initiation and up-to-date rates have also been consistently lower for Medicaid enrollees and the uninsured, as compared to the privately insured [5–7]. For example, in 2013, up-to-date rates across demographic groups, except for Hispanics, the only group in which this finding was reversed, were 6–17 percentage points lower among U.S. Medicaid enrollees ages 50–64 than the privately insured in the same age group [5].

The objective of this paper is to assess associations between the timing of national and state policies and incident CRC screening behaviors among newly enrolled Medicaid beneficiaries turning 50 between 2010 and 2014. We focus specifically on CRC screening in Oregon because screening rates in this state closely resemble national rates; Oregon data for 2014 show that 60.8% of adults ages 50–64 (vs. 60.8% nationally) and 78.4% of adults 65 and older (vs. 76.1% nationally) were up-to-date on CRC screening [4]. In addition, Oregon enacted multiple policies designed to increase access to insurance and preventive services during these years, providing an opportunity to compare incident screening annually in the context of health policy changes. We first develop a timeline of state and federal policy changes related to Medicaid coverage and preventive services during the study period. We then explore changes in CRC screening initiation, defined as screening within 12 months after an enrollee turned 50 years old, based on year enrolled in Medicaid and year becoming age eligible (i.e., turned 50). Screening initiation was studied because it is an important prevention milestone, and an age at which screening in a single year can be assessed for guideline concordance.

## Methods

**Fig. 1:**
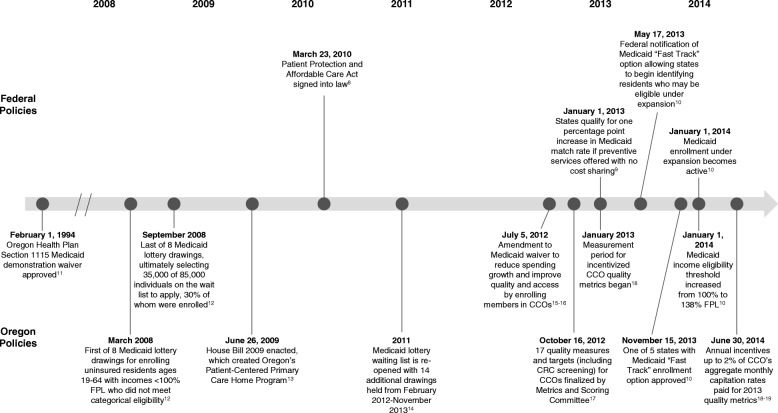
Timeline of Medicaid expansion and preventive services coverage in Oregon and nationally

We conducted a policy review and retrospective claims analysis to understand incident CRC screening in Oregon between 2010 and 2014 within the Medicaid population. First, we reviewed the literature to identify national and Oregon-specific policies implemented between 2010 and 2014 aimed at either increasing access to Medicaid coverage or improving CRC screening among Medicaid enrollees. Second, we consulted with policy experts to ensure that our list of policies was comprehensive and accurate. Figure 1 summarizes relevant policy changes such as, at the national level, the enactment of the Affordable Care Act (ACA) in 2010 and increased Medicaid matching rates for states offering preventive services with no cost sharing in 2013 [8–19]. Oregon policy changes included the enrollment of Medicaid members into coordinated care organizations (CCOs, a type of accountable care organization) in 2012, the measurement of CRC screening as a CCO quality incentive beginning in 2013, and the expansion of the state’s Medicaid program in 2014.

We then obtained Oregon Medicaid claims data from 2010 to 2015 (Additional file 1: Figure S1) to determine if there was a differential impact of first gaining Medicaid coverage and/or becoming age-eligible for CRC screening in any given year that may be associated with the respective policy changes. For this sample, we included individuals who turned age 50 from 2010 to 2014 and were continuously enrolled in Medicaid for at least 11 of 12 months following their 50th birthday. We excluded enrollees with a birthday during the last quarter of each study year to assess CRC screening initiation over a 12-month period consistently across all study years, given the transition from ICD-9 to ICD-10 codes in October 2015. We also excluded enrollees with a history of CRC, colectomy, or end-stage renal disease (Additional file 1: Table S1); those who passed away during the study period; and those dually eligible for Medicaid and Medicare because their patterns of care are different than those insured solely by Medicaid [7] and because we lacked access to Medicare claims.

Our primary outcome was any indication of CRC screening with colonoscopy, sigmoidoscopy, or stool testing within 12 months after turning 50 (Additional file 1: Table S2). Using claims data alone, it is difficult to identify guideline-concordant CRC screening due to the multiple ways in which people can become up-to-date on screening (e.g. colonoscopy 10 years ago, stool testing in the past year) as well as fluctuations in coverage for Medicaid enrollees over time (e.g., screening completed while a patient is privately insured will not appear in Medicaid claims). Therefore, we focused on this newly age-eligible Medicaid population who turned 50 during the study window to minimize the chance of any missed CRC screenings in our dataset.

Finally, we used a log Poisson model to assess whether eligible Medicaid enrollees were more likely to initiate CRC screening within 12 months after their 50th birthday in response to national and/or state policy changes within the year that they turned 50 (e.g., ACA Medicaid expansion). In this way, turning 50 in a particular policy year is a form of random assignment. We report screening rates for years 2011, 2012, 2013, and 2014, with 2010 serving as the reference group for this analysis. In addition, we consider differences in CRC screening among these 50-year-old enrollees as a result of policy changes in the same year in which they first enrolled in Medicaid. We defined new enrollment in Medicaid as having no enrollment from 2005 until the year an individual turned 50. All analyses controlled for potential covariates, including sex, race/ethnicity, geography, distance to endoscopy facility, having a chronic condition, visiting a primary care provider (PCP), and enrolling in Medicaid and turning 50 in the same year. Similar to prior studies, distance to endoscopy facility was calculated using zip code centroids to determine the closest distance from the member’s residence to the nearest endoscopy facility [6], and PCP visit was defined as any visit with a primary care procedure code in the calendar year [20]. Analyses were conducted using R, version 3.4.4. We assessed statistical significance using an alpha level of 0.05 and report 95% confidence intervals (CI).

## Results

A total of 14,576 Oregon Medicaid enrollees turned 50 between 2010 and 2014 and were newly age-eligible for CRC screening (Table 1). Eligible enrollees included urban (57%) and rural (43%) residents, 56% were female, 72% were white, and 10% were Hispanic. Most (78%) enrollees lived within 5 miles of the nearest endoscopy facility. Three-quarters (78%) of the enrollees visited their PCP during the index year, and 73% had a chronic condition. One in four (26%) individuals enrolled in Medicaid for the first time in 2014; 60% first enrolled by 2010. The year 2014 was when most (44%) enrollees turned 50. Of these individuals, 2429 (17%) completed any form of CRC screening within 12 months after their 50th birthday.

**Table 1:** Characteristics of Oregon Medicaid enrollees who turned age 50 in 2010–2014

Characteristic	Total Population (%, N)	Completed CRC Screening Within 12 Months After 50th Birthday (%, N)
Total (N) Sex	14,576	2429
Male	44.4 (6479)	42.0 (1019)
Female	55.6 (8097)	58.0 (1410)
Race/Ethnicity		
White	71.8 (10,469)	71.4 (1735)
Hispanic	10.3 (1497)	9.1 (220)
African American	3.9 (568)	5.2 (127)
Other/Unknown	14.0 (2042)	14.3 (347)
Geography		
Rural	43.0 (6274)	39.9 (969)
Urban	57.0 (8302)	60.1 (1460)
Distance to endoscopy facility		
> 5 miles	22.4 (3263)	20.4 (496)
≤ 5 miles	77.6 (11,313)	79.6 (1933)
Chronic condition		
No	26.7 (3888)	11.0 (268)
Yes	73.3 (10,688)	89.0 (2161)
Primary care provider visit		
No	21.9 (3186)	4.0 (98)
Yes	78.1 (11,390)	96.0 (2331)
Year turned 50		
2010	10.5 (1529)	9.5 (231)
2011	15.3 (2230)	15.4 (373)
2012	15.6 (2276)	16.0 (388)
2013	14.4 (2095)	14.1 (342)
2014	44.2 (6446)	45.1 (1095)
Year first enrolled in Medicaid		
2010 or earlier	59.9 (8738)	58.6 (1423)
2011	8.8 (1289)	8.7 (212)
2012	3.6 (527)	3.6 (87)
2013	1.9 (277)	2.6 (64)
2014	25.7 (3745)	26.5 (643)

Table 2 presents risk ratios (RR) to identify how much more likely Medicaid enrollees were to initiate CRC screening within 12 months after turning 50 based on demographics, geographic characteristics, health status, healthcare utilization, year turned 50, and year enrolled in Medicaid. Newly age-eligible Medicaid enrollees who had a PCP visit were significantly more likely to get screened than those without a visit (RR: 5.38; 95% CI = 4.33, 6.70). Enrollees with one or more chronic conditions were 1.66 times more likely to initiate screening within 12 months than those without (95% CI = 1.45, 1.91). Compared to those who first enrolled in 2010, those who first enrolled in Medicaid in 2013 were 1.58 times as likely to get screened (95% CI = 1.20, 2.09), and first time enrollees in 2014 were 1.31 times as likely to get screened (95% CI = 1.15, 1.49). Although the RR was higher in 2013 relative to 2014, the absolute number of individuals who enrolled in Medicaid in 2014 was considerably higher than in 2013. In terms of race and ethnicity, Hispanics were 1.16 times as likely to initiate CRC screening as white enrollees (95% CI = 1.00, 1.34). Sex, rural/urban residence, distance from an endoscopy facility, year turned 50, and turning 50 and enrolling in Medicaid in the same year prior to Medicaid expansion were not statistically significant.

**Table 2:** Relative risk of completing CRC screening among Oregon Medicaid enrollees who turned age 50 in 2010–2014 within 12 months after 50th birthday

Characteristic	Risk Ratio (95% CI)	*P*-value
Sex		
Male	Reference	
Female	1.03 (0.95–1.11)	.548
Race/Ethnicity		
White	Reference	
Hispanic	1.16 (1.00–1.34)	.044
African American	1.19 (0.98–1.43)	.075
Other/Unknown	1.10 (0.98–1.24)	.121
Geography		
Rural	Reference	
Urban	1.08 (0.96–1.22)	.190
Distance to endoscopy facility		
> 5 miles	Reference	
≤ 5 miles	1.05 (0.94–1.19)	.391
Chronic condition		
No	Reference	
Yes	1.66 (1.45–1.91)	<.0001
Primary care provider visit		
No	Reference	
Yes	5.38 (4.33–6.69)	<.0001
Year turned 50		
2010	Reference	
2011	1.13 (0.96–1.34)	.143
2012	1.12 (0.95–1.33)	.163
2013	1.05 (0.88–1.24)	.610
2014	1.11 (0.94–1.31)	.207
Year first enrolled in Medicaid		
2010 or earlier	Reference	
2011	1.05 (0.90–1.24)	.509
2012	1.11 (0.88–1.41)	.369
2013	1.58 (1.20–2.09)	.001
2014	1.31 (1.15–1.49)	<.0001
Turned 50 and first enrolled in Medicaid in same year (2010–2013)		
No	Reference	
Yes	0.98 (0.77–1.25)	.871

## Discussion

In this analysis, we explored the potential impact of national and state policies on the likelihood of CRC screening initiation in newly age-eligible Medicaid enrollees between 2010 and 2014 in Oregon. Compared to 2010, we found a statistically significant increase in CRC screening initiation among individuals who first enrolled in Medicaid in either 2013 or 2014. These results mirror national CRC screening trends, in which CRC screening plateaued from 2010 to 2013, followed by an increase from 2013 to 2015 [21]. Mapping these trends onto state and federal policy changes provides an opportunity to understand how health policy may drive screening initiation behaviors.

The uptake in CRC screening among those who first enrolled in 2013 and 2014 is associated with the enactment of multiple policies intended to increase Medicaid enrollment and coverage of preventive services within this population during this timeframe. Both federal and state policies during this period incentivized CRC screenings. For example, in 2012 Oregon assigned Medicaid members into newly formed CCOs [22], in which CRC screening began to be measured as a quality metric in 2013 with gradually increasing financial incentives [18, 19]. Meanwhile, the expansion of Oregon’s Medicaid program in 2014, and the decision to participate in “fast track” enrollment in which the state could start to identify and begin outreach in 2013 to residents who would become newly eligible for coverage through Medicaid expansion, provided more state residents with access to coverage [10]. The larger increase in the number of new enrollees screened in 2014 compared to 2013, along with the finding that the year in which individuals turn 50 is not statistically significant, point to differences in CRC screening behaviors among those newly enrolled through Medicaid expansion compared to those newly enrolled in prior years.

While prior research [23, 24] demonstrates that insurance access is an important predictor of CRC screening, we also observed differences in CRC screening initiation based on patient characteristics. This suggests that, in addition to increased coverage, certain groups may need more assistance in completing recommended preventive care. Within our population, PCP visits and the presence of chronic conditions were the biggest determinants of CRC screening within 12 months. Individuals who visit their PCP or who have a chronic condition may be more actively engaged in their health and more likely to get screened than those who have not seen their PCP in the calendar year or who do not have a chronic condition [6, 25, 26]. Given efforts to promote CRC screening through policy in Oregon, providers may have been more likely to recommend screening during the study window. Clinics and facilities may also have increased the use of various screening modalities beyond colonoscopy to achieve the CCO quality metric [20, 27]. For example, following the development of Oregon’s Patient-Centered Primary Care Home Program (PCPCH), Oregon PCPs were focused on improving their delivery of preventive care and PCPCHs received training related to their care delivery and metric-specific efforts [13]. In contrast, Medicaid enrollees who were assigned to a CCO and provider but did not establish care right away likely did not receive a recommendation for CRC screening. Additional resources and population outreach strategies may be needed to meet the growing demand of newly enrolled Medicaid members.

The CRC screening rate was also higher among Hispanics – a pattern that has mixed prior evidence. While national CRC screening rates are lower among Hispanics compared to non-Hispanic whites [28, 29], research has shown relatively high CRC screening rates among Hispanics who are insured or English-speaking [30–33]. Moreover, Hispanics who are included in mailed FIT outreach programs are found to have higher FIT completion rates than non-Hispanic whites [34]. In Oregon, the strategies employed by CCOs to improve equity, such as the use of community health workers, have improved primary care use and screening uptake among racial and ethnic groups [35]. In addition, an ongoing Oregon statewide public health campaign intended to promote CRC screening, initially piloted in 2011, also provides dedicated resources to reach Spanish-speaking residents as well as other vulnerable groups, including African Americans, Native Americans, and rural communities [36]. Although this study did not assess differences in screening modality, the higher CRC screening among Hispanics may also reflect the trend in increased use of fecal testing during the study period in Oregon [20]; notably, fecal testing has been shown to be an effective and acceptable screening modality among Hispanics [37, 38]. Nevertheless, there is a continued need for targeted policies to reduce disparities in preventive care.

There are several strengths of this analysis. We developed a comprehensive timeline of federal and Oregon-specific health policy changes during the study window that had the potential to influence CRC screening patterns. We also identified opportunities to improve CRC screening among Medicaid beneficiaries, a population with known disparities in use of recommended preventive services as well as CRC outcomes.

This analysis includes some limitations. First, we anticipate that CRC screening initiation is underestimated based on our use of claims data alone; however, claims analyses provide a validated method of identifying screening patterns over multiple years without the potential biases (e.g. recall bias) associated with self-reported data collection [39, 40]. Second, the impact of the evaluated health policies may also be muted because we do not know the percentage of unscreened individuals who initiated screening soon after the 12-month window, and it may take longer to see the effects of Medicaid expansion on preventive screening rates. In addition, the year 2010 is somewhat different than the other study years because it included individuals who first enrolled in 2010 and those who enrolled in prior years. Finally, while our data point to a cumulative positive effect of the related health policies, we are unable to separate out the individual impact of specific policy interventions or assess causality. Despite these limitations, this analysis provides a snapshot of CRC screening uptake among a vulnerable population during a period characterized by numerous policy changes.

Continued assessment of CRC screening rates among individuals new to Medicaid and newly turned 50 could be helpful in identifying longer-term trends of national and state policies. Since 2014, new statewide policies have gone into effect in Oregon that further promote CRC screening across insurers. These policies include House Bill 4085, which eliminates cost sharing for recommended CRC screening tests, inclusive of the removal of polyps during screening [41], and House Bill 2560, which provides coverage of diagnostic colonoscopies following an abnormal fecal test result among adults at least 50 years of age.^42^ It will be important to evaluate the impact of these and future policy efforts to facilitate CRC screening in the larger state population and among Medicaid enrollees. Findings may also inform other states that are exploring opportunities to improve CRC prevention by expanding access to Medicaid and incentivizing screening.

## Conclusions

We observed higher CRC screening initiation in newly age-eligible Oregon Medicaid enrollees during a time period when insurance expansion and policies to promote preventive service use were implemented. Being newly enrolled in Medicaid in 2013 and 2014 was significantly associated with increased CRC screening initiation. In this insured population, PCP visits and presence of a chronic condition were particularly strong indicators for CRC screening. Our findings show that recent state and national health promotion policies appear to have supported CRC screening among Medicaid enrollees. Such policies, combined with facilitators at the practice- and patient-levels, are important for promoting CRC screening uptake.

## Additional file

Additional file 1: **Figure S1.** Inclusion and exclusion criteria. **Table S1.** Billing codes indicating CRC screening procedures. **Table S2.** Billing codes indicating exclusion criteria (DOCX 34 kb)

## Abbreviations

ACA: Affordable Care Act
CCO: Coordinated Care Organization
CI: Confidence Interval
CPT: Current Procedural Terminology
CRC: Colorectal Cancer
FIT: Fecal Immunochemical Test
HCPCS: Healthcare Common Procedure Coding System
ICD-10: International Classification of Diseases, Tenth Edition
ICD-9: International Classification of Diseases, Ninth Edition
PCP: Primary Care Provider
PCPCH: Patient-Centered Primary Care Home Program
RR: Risk Ratio
