# Supplementary material for: Colorectal cancer screening in newly insured Medicaid members: a review of concurrent federal and state policies
Source: BMC Health Serv Res. 2019 May 9;19:298. doi: 10.1186/s12913-019-4113-2 (PMC6509857; doi:10.1186/s12913-019-4113-2)
Supplement: Supplementary file 1 — Figure S1. Inclusion and exclusion criteria. Table S1. Billing codes indicating CRC screening procedures. Table S2. Billing codes indicating exclusion criteria (DOCX 34 kb) [file 12913_2019_4113_MOESM1_ESM.docx]

**Appendix Figure A1. Inclusion and exclusion criteria**

Unique records in Medicaid claims

**1,701,185**

Turned 50 years old during study period

**72,946**

Continuously enrolled at least 11 out of 12 months following 50^th^ birthday

**28,421**

Alive through study period

**27,685**

Not dually eligible for Medicare

**20,677**

No history of CRC or total colectomy

**20,637**

No history of end-stage renal disease

**20,578**

Turned 50 in the first 9 months of the year

**14,576**

Did not turn 50 during study period

**1,628,239**

Not continuously enrolled at least 11 out of 12 months following 50^th^ birthday

**44,525**

Deceased by end of study period

**736**

Dually eligible for Medicare

**7,008**

History of CRC or total colectomy

**40**

History of end-stage renal disease

**59**

Did not turn 50 in the first 9 months

**6,002**

**Appendix Table A1. Billing codes indicating exclusion criteria**

| **Reason for exclusion** | **Codes** |
| --- | --- |
| Colorectal cancer | **HCPCS**: G0213, G0214, G0215, G0231  **ICD-9 Diagnosis**: 153, 154.0, 154.1, 154.2, 154.3, 197.5, V10.05, V10.06 |
| Total colectomy | **CPT**: 44150, 44151, 44152, 44153, 44155, 44156, 44157, 44158, 44210, 44211, 44212  **ICD-9 Procedure**: 45.8, 45.81, 45.82, 45.83 |
| End stage renal disease | **ICD-9 Diagnosis:** 585.6 |

Abbreviations: ICD-9-CM = International Classification of Diseases, 9^th^ Edition, Clinical Modification, CPT = Current Procedural Terminology, HCPCS = Healthcare Common Procedure Coding System.

**Appendix Table A2. Billing codes indicating CRC screening procedures**

| **Screening test modality** | **Codes** |
| --- | --- |
| Fecal occult blood test (FOBT) or fecal immunochemical test (FIT) | **CPT**: 82270 (FOBT), 82274 (FIT), 82272*, 82273*, 82274*  **HCPCS**: G0328 (FIT), G0107 (FIT) |
| Colonoscopy | **CPT**: 44388, 44389, 444390, 44391, 44392, 44393, 44394, 44397, 45355, 45378, 45379, 45380, 45381, 45382, 45383, 45384, 45385, 43386, 45387, 45391, 45392  **HCPCS**: G0105, G0121  **ICD-9 Procedure**: 45.21, 45.22, 45.23, 45.25, 45.41, 45.42, 45.43, 48.36 |
| Flexible sigmoidoscopy | **CPT**: 45300, 45303, 45305, 45307, 45308, 45309, 45315, 45317, 45320, 45321, 45327, 45330, 45331, 45332, 45333, 45334, 45335, 45337, 45338, 45339, 45340, 45341, 45342, 45345  **HCPCS**: G0104  **ICD-9 Procedure**: 45.24, 48.21, 48.22, 48.23, 48.24 |

Abbreviations: ICD-9-CM = International Classification of Diseases, 9^th^ Edition, Clinical Modification, CPT = Current Procedural Terminology, HCPCS = Healthcare Common Procedure Coding System. *Indicates a non-specific code for fecal testing.
